# Terminal Residues and Risk Assessment of Spiromesifen and Spirodiclofen in Tomato Fruits

**DOI:** 10.3390/plants12071493

**Published:** 2023-03-29

**Authors:** Osama I. Abdallah, Rania M. Abd El-Hamid, Nevein S. Ahmed, Sayed M. Saleh, Fahad M. Alminderej

**Affiliations:** 1 Department of Pesticide Residues and Environmental Pollution, Central Agricultural Pesticide Laboratory (CAPL), Agricultural Research Center (ARC), Giza 12618, Egypt; 2 Department of Chemistry, College of Science, Qassim University, Buraidah 52571, Saudi Arabia; 3 Chemistry Branch, Department of Science and Mathematics, Faculty of Petroleum and Mining Engineering, Suez University, Suez 43721, Egypt

**Keywords:** dissipation kinetics, LC-MS/MS, method validation, risk assessment, spirodiclofen, spiromesifen

## Abstract

Insecticides are important to increase crop yields, but their overuse has damaged the environment and endangered human health. In this study, residues of spiromesifen and spirodiclofen were determined in tomato fruit using a simple and efficient analytical procedure based on acetonitrile extraction, extract dilution, and UPLC-MS/MS. The linearity range was 1–100 µg/kg and 0.5–100 µg/kg, and the correlation coefficient (R^2^) and residuals were ≥0.9991 and ≤16.4%, respectively. The limit of determination (LOD) was 0.26 and 0.08 µg/kg, while the limit of quantification (LOQ) was verified at 5 µg/kg. The relative standard deviation of spiked replicates at 5 µg/kg analyzed in one day (RSDr, n = 6) was ≤8.35%, and within three different days (RSDR, n = 18) it was ≤15.85%, with recoveries exceeding 91.34%. The method recovery test showed a satisfactory value of 89.23–97.22% with an RSD of less than 12.88%. The matrix effect was determined after a 4-fold dilution of the raw extract and was −9.8% and −7.2%, respectively. The validated method was used to study the dissipation behavior of the tested analytes in tomato fruit under field conditions. First-order kinetics best described the dissipation rates. The calculated half-lives were 1.49–1.83 and 1.91–2.38 days for spiromesifen and spirodiclofen, respectively, after application of the authorized and doubled authorized doses, indicating that spiromesifen dissipated more rapidly than spirodiclofen. The final residue concentrations of spiromesifen and spirodiclofen were 0.307–0.751 mg/kg and 0.101–0.398 mg/kg, respectively, after two or three applications, and were below the European Union (EU) maximum residue limits. The chronic risk assessment indicates that both insecticides are safe for adult consumers.

## 1. Introduction

Tomato fruit (*Lycopersicon esculentum* Mill.) is one of the most widely grown crops in the world. Egypt is a major producer and consumer of tomatoes, ranking sixth among producing countries with an annual production of 6.7 million tons [1]. Raw tomatoes and their products have numerous health benefits. They contain lycopene, antioxidants, tomatine, calcium, niacin, and vitamins A, C, and E [2]. Therefore, they have a significant impact on human health by reducing the risk of cancer and chronic degenerative diseases [3,4,5,6]. Many insect pests can attack tomatoes during the growing season, so controlling these insects can be difficult as they become lodged in the shoots and fruits. Spray insecticides are effective, but their continued use can promote resistance [7]. The residues left behind can lead to acute and chronic health hazards and affect the nutritional quality of tomatoes [8].

**Figure 1:**
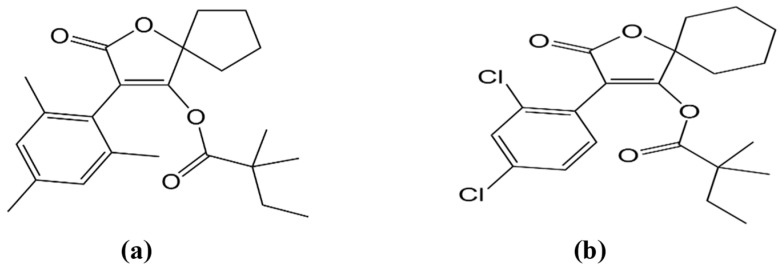
Chemical structure of spiromesifen (**a**) and spirodiclofen (**b**).

Spiromesifen [2-oxo-3-(2,4,6-trimethylphenyl)-1-oxaspiro[4.4]non-3-en-4-yl-3,3-dimethylbutanoate] and spirodiclofen [3-(2,4-dichlorophenyl)-2-oxo-1-oxaspiro[4.5]dec-3-en-4-yl-2,2-dimethylbutyrate] (Figure 1a,b) are novel insecticides that fall into the category of spirocyclic phenyl-substituted tetronic acid derivatives and act as acetyl-coenzyme carboxylase (ACCase) inhibitors by disrupting lipid biosynthesis at all developmental stages of mites that infest tomatoes, chili, eggplant, cotton, and other crops [9]. Because spiromesifen and spirodiclofen are relatively new insecticides/acaricides, few data are available on residues and rates of dissipation in vegetables and fruits under field conditions: chili [10], okra [11], cabbage [12], eggplant [13], tomatoes [14], and citrus [15,16].

In the last 20 years, numerous extraction methods have been presented for pesticide residue analysis based on acetonitrile, acetone, or ethyl acetate as extraction solvents. Due to its remarkable selectivity and specificity, mass spectrometry (MS) can be used with liquid chromatography (LC) for residue determination. Co-eluted interferences in samples may not be a problem [17,18,19]. Many laboratories have used “Quick, Easy, Cheap, Effective, Rugged, and Safe” (QuEChERS) methods with good results and high recoveries [20,21,22,23,24].

The objective of this study was to: (1) validate an effective, inexpensive, and simple method for the simultaneous determination of spiromesifen and spirodiclofen residues in tomato using the validation criteria of linearity, LOD, LOQ, trueness (recovery %), precision (RSD %), and matrix effect (ME %), (2) evaluate the rate of dissipation and determine the half-lives of the target insecticides in tomatoes grown under field conditions, (3) determine the final residues after multiple applications, and (4) assess the risks that may result from long-term consumption of tomatoes (chronic risk) and propose a pre-harvest interval (PHI). This research will provide information for developing strategies to safely use spiromesifen and spirodiclofen in tomatoes to avoid health problems for consumers.

## 2. Results and Discussion

### 2.1. LC-MS/MS Optimization

The MRM transition and associated parameters were tuned for optimizing the parameters of the MS/MS spectrometer and for achieving a high sensitivity of the tested analytes, using a reference solution of 500 µg/L each in a positive electro spray ionization (ESI) mode. The high-flow infusion technique was used to optimize collision and cone voltage using a Harvard pump (South Natick, MA, USA) in continuous injection mode. To meet the performance criteria for identification, two ions were selected and the ion with a lower intensity was chosen as the qualifier, whereas that with the highest intensity was chosen as the quantifier [25]. Table 1 provides a list of the optimized MS/MS parameters.

The mobile phase component was selected based on tests carried out using the commonly used organic solvents methanol or acetonitrile, and water was used as the main component for reversed-phase chromatography. The constituents of water/methanol or water/acetonitrile were tested in gradients, as mentioned in the above section, LC-MS/MS. It was found that the use of acetonitrile/water as a mobile phase component decreased the signal sensitivity of spiromesifen and spirodiclofen by 30% and 2182%, respectively, compared to methanol/water. Therefore, methanol/water was chosen as the component of the mobile phase. The addition of formic acid to the mobile phase at a concentration of 0.1% resulted in a significant (*p* ˃ 0.05) decrease in sensitivity of 215% and 217%, respectively.

### 2.2. Effect of Dilution on the Matrix Effect

It was found that co-extracts eluted with spiromesifen and spirodiclofen suppressed signal sensitivity by 35% and 43.6%, respectively, when analyzed directly without cleanup compared with signal sensitivity in pure acetonitrile.

The use of the PSA adsorbent at 25 mg/mL resulted in a significant (*p* < 0.05) reduction in signal sensitivity of 29.6% and 35.2%, respectively, compared with pure acetonitrile. A previous study reported that analysis with LC-MS/MS may cause a negligible matrix effect [17,18,19]. This was the case because gradient elution was performed with a long run time and high ratios of water content in the initial gradient program that allowed the separation of co-extracted materials in the column and before ionization. In our study, we challenged the separation of the target analyte in a short run time by using a high ratio of organic solvent at the beginning of the gradient to accelerate the elution of the analytes, so the co-eluted materials were increased during the ionization step and suppressed the ion sensitivity of the target analytes.

**Figure 2:**
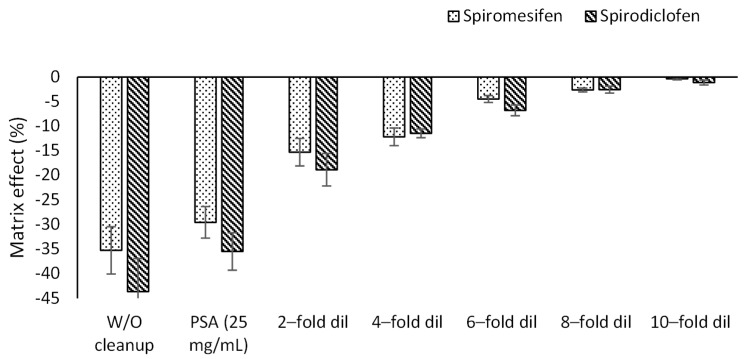
Effect of using PSA (25 mg/mL) vs. dilution (2–10 fold) on the matrix effect.

Therefore, the effect of dilution of the raw extract compared to using the PSA adsorbent and the raw extract (without cleanup) was investigated to minimize the effects of the co-extracted compounds on signal sensitivity. The resulting data (Figure 2) show that a 2-fold dilution resulted in a significant increase in peak sensitivity of spiromesifen compared with raw extract (W/O cleanup) and the raw extract purified with PSA, in addition to a nonsignificant suppression of signal sensitivity (−15.3%) compared with pure acetonitrile. In contrast, 4-fold dilution resulted in a nonsignificant (*p* > 0.05) suppression of spirodiclofen signal sensitivity of −12.2% compared with that prepared in pure acetonitrile, and a significant (*p* < 0.05) increase in signal sensitivity compared with that prepared in raw extract or extract cleaned by PSA. Further dilution up to 10-fold of the raw extract was not significantly different (*p* > 0.05) from 2-fold and 4-fold dilution of the raw extract for spiromesifen and spirodiclofen, respectively. Therefore, the 4-fold dilution was selected for further analysis.

### 2.3. Method Validation

#### 2.3.1. Linearity

The linearity range, residuals, and correlation coefficients (R^2^) were tested using the least squares method to measure the range within analyte concentrations directly proportional to detector response. Linearity ranged from 1–100 µg/kg and from 0.5 to 100 µg/kg with correlation coefficients (R^2^) of 0.9993 and 0.9991 and residuals of 16.4% and 13.2% for spiromesifen and spirodiclofen, respectively (Table 2).

#### 2.3.2. LOD and LOQ

The lowest fortified concentration at which a signal-to-noise (S/N) ratio of 3 was achieved at or near the retention time of the peaks of interest with acceptable accuracy is defined as the LOD. The LODs for spiromesifen and spirodiclofen were calculated as 0.26 µg/kg and 0.08 µg/kg, respectively. The lowest spiked value that achieves acceptable accuracy is defined as the LOQ. The 5 µg/kg value for spiromesifen and spirodiclofen resulted in good recoveries and precisions of 89.23–93.52% and 5.87–6.22%, respectively, and is considered the LOQ. The reported LOQs for spiromesifen and spirodiclofen are 200- and 100-fold lower than the European Commission MRLs of 1 mg/kg and 0.5 mg/kg, respectively (Table 2).

#### 2.3.3. Accuracy

The accuracy of the methods was evaluated in terms of precision and recovery. For precision, relative standard deviation (RSD) values were determined for replicates at 5 µg/kg concentrations. Precision, expressed as intra-day (RSDr, n = 6, on one day) and inter-day repeatability (RSDR, n = 18, on three different days), was 8.35% and 15.85%, respectively, for spiromesifen and 6.68% and 11.74%, respectively, for spirodiclofen, with mean recoveries ranging from 91.34% to 96.42%, indicating good precision of the method (Table 2).

#### 2.3.4. Recovery

The average recoveries of spiromesifen and spirodiclofen at four spiking levels of 0.01, 0.01, 1, and 5 mg/kg in tomato fruit were 90.75–97.22% and 89.23–96.71%, with RSD less than 8.78% and 12.88, respectively (Table 3).

The results are in agreement with the SANTE guide for pesticide residues, which states that the recovery values for each spiking should be between 70 and 120%, with an RSD of less than 20% [25].

#### 2.3.5. Matrix Effect (ME)

**Figure 3:**
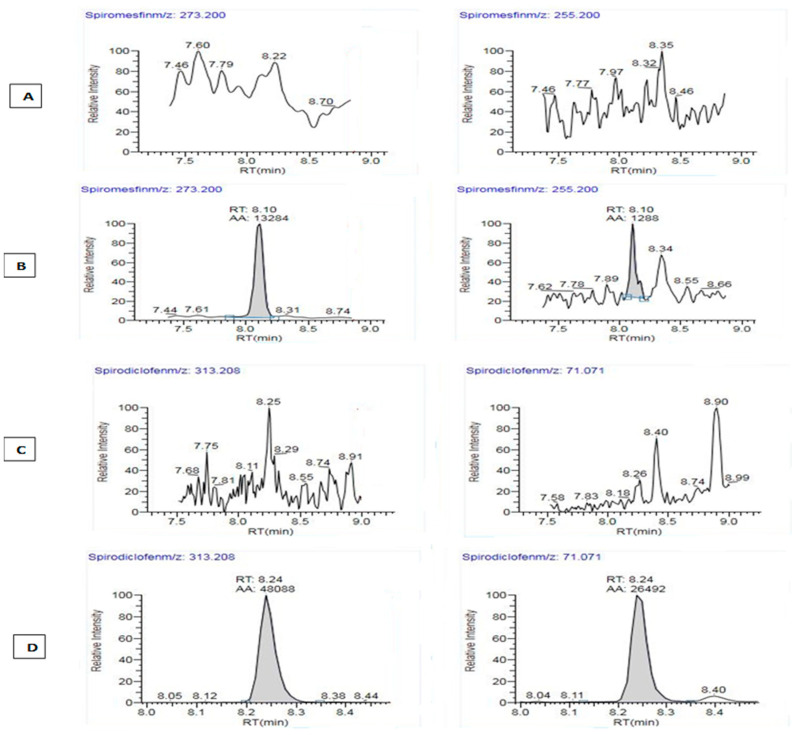
LC/MS/MS representative chromatograms of blank sample extract (**A**,**C**), spiromesifen fortified sample extract (5 µg/kg) (**B**), spirodiclofen fortified sample extract (5 µg/kg) (**D**).

The signal enhancement or suppression was investigated by comparing the slope of matrix-matched calibration curve to slope obtained from the in-solvent calibration curve using the formula ME = ((slope-M/Slope-S) − 1) ∗ 100 [26,27,28]. The MEs for spiromesifen and spirodiclofen were −8.9% and −7.2%, respectively, indicating that the final extract from tomato fruit after 4-fold dilution non-significantly suppressed the ionic responses of both insecticides (Table 2). Endogenous substances, such as fatty acids, carbohydrates, and pigments, may have been insufficiently removed, resulting in suppression [29]. Despite the lower matrix effect, matrix-matched calibration curves were used to reduce ME and to provide accurate results. Figure 3 shows a representative chromatogram of spiromesifen and spirodiclofen.

### 2.4. Dissipation of Spiromesifen and Spirodiclofen in Tomato Fruits

**Figure 4:**
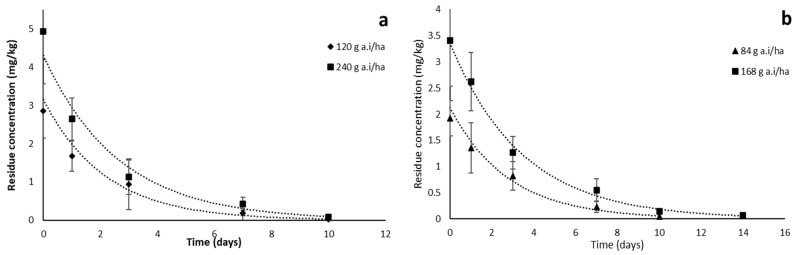
Dissipation of spiromesifen (**a**) and spirodiclofen (**b**) in tomato fruits.

The dissipation profiles of spiromesifen and spirodiclofen residues in tomato fruit were observed up to 14 days after spraying the authorized dose of 120 g a.i/ha and 84 g a.i/ha and the double dose of 240 g a.i/ha and 168 g a.i/ha, respectively. The results showed that the residues were rapidly degraded during the first days. The initial residues of spiromesifen were 2.854 and 4.935 mg/kg after application of the authorized and double dose, respectively. Spiromesifen was 67–77% dissipated after 3 days. Residues were 0.192 and 0.427 mg/kg 7 days after spraying. After 10 days of spraying, residues were 0.022 and 0.134 mg/kg. These residues were below the MRL of 1 mg/kg (EU-MRL database). For spirodiclofen, the initial residues were 1.921 and 3.412 mg/kg; it was shown that spirodiclofen dissipated slowly in tomato fruit. Residues decreased to 57–63% 3 days after application. After 10 days, residues were below the MRL of 0.5 mg/kg (EU-MRL database) (Figure 4).

### 2.5. Half-Life and PHI

After closely examining the correlation co-efficient (r^2^), first-order kinetics proved to be the best model for describing the dissipation rates of spiromesifen and spirodiclofen. The dynamic equations for the dissipation of spiromesifen and spirodiclofen in tomato fruit after application at the authorized dose and double the authorized dose were C_t_ = 3.1593 e^−0.463x^ and C_t_ = 4.3086 e^−0.379x^, and C_t_ = 2.1263 e^−0.362x^ and C_t_ = 3.3649 e^−0.291x^, and the estimated t_1/2_ were 1.49 and 1.82 days, and 1.91 and 2.38 days, respectively (Table 4).

Because the octanol/water partition coefficient (Logk_ow_) of spiromesifen and spirodiclofen are 4.55 and 5.83, respectively, they are relatively hydrophobic and poorly soluble in water, so they penetrate the outer layer of the exocarp (outer layer) and penetrate a little into the mesocarp (fleshy interior). However, most of the residues remained in the exocarp. In addition, the vapor pressure of spiromesifen, 1.5 × 10^−4^ mmHg, is higher than that of spirodiclofen, 5.25 × 10^−9^ mmHg (https://pubchem.ncbi.nlm.nih.gov/, accessed on 1 March 2023). Still, this factor plays a minor role in interpreting our results because the application doses are different and, therefore, the initial deposits differ. The stability of the six-ring carbon cycle in spirodiclofen compared with the five-ring carbon cycle in spiromesifen (Figure 1) might have been stabilizing, which might have played a significant role in the rapid dissipation of spiromesifen.

For spiromesifen, the current results were roughly comparable with half-lives of 0.93–1.38 days for tomato [30], 1.6, 1.8, 1.9, and 1.7 days for okra, bell pepper, chili, and brinjal, respectively [31], and 1.32–2.18 days for brinjal [32]. In contrast, a few reports have investigated the longer half-life of spiromesifen in different crops: 5.5–6.2 days, 2.52–2.88 days, and 5.0–8.5 days in apple, eggplant, and leaf tea, respectively [13,33,34,35], and 6–6.5 days in tomato [14]. For spirodiclofen, on the other hand, little data are available on crop dissipation kinetics. Wang et al. (2020) reported a half-life of 14.1 days when spirodiclofen was applied to kumquat covered with a plastic film [36]. Sun et al. (2013) also reported a long half-life of 6.5 to 13.5 days when spirodiclofen was sprayed on citrus at different sites in China [15]. In contrast, Morsy and EL-Hefny (2017) reported a shorter half-life of 0.91 days when spirodiclofen was applied to apple fruit [37]. The variations in half-life could be attributed to varying climatic conditions, crop types, application doses, formulation types, and stages at which the application was conducted [38].

The pre-harvest intervals (PHI) are the periods between the last application and harvest that allow pesticide residues in harvested crops to fall to maximum residue levels (MRLs). The proposed PHI was 2.48 and 3.85 days for spiromesifen, and 3.99 and 6.55 days for spirodiclofen, when the authorized dose and double the authorized dose were applied, respectively.

### 2.6. Terminal Residues

Spiromesifen and spirodiclofen were sprayed two and/or three times, seven days apart at the authorized dose and at double the authorized dose. The results in Table 5 show that the concentrations of the target insecticides decreased with increasing sample intervals, probably related to the degradation of the target analytes and the development of the tomato fruit. After 7 days, the tomato fruits were harvested, and the mean residue levels of spiromesifen and spirodiclofen were below the MRL of 1 mg/kg and 0.5 mg/kg after two and/or three treatments, respectively. Residues of spiromesifen were 0.471–1.652 mg/kg and 0.168–0.757 mg/kg at PHI of 3 and 7 days, respectively, after the authorized and double doses were applied. Similarly, spirodiclofen residues were 0.585–1.414 mg/kg and 0.101–0.398 mg/kg at PHI of 3 and 7 days, respectively. At the end of the sampling periods, residues of spirodiclofen were higher than those of spiromesifen, which may be due to the higher application dose of spirodiclofen (120 g a.i/ha and 240 g a.i/ha). These results were included in a dietary risk assessment study to investigate the risk of tomato fruit sprayed with the tested insecticides.

### 2.7. Chronic Dietary Risk Assessment

In response to public concern about pesticide residues in crops, the WHO/FAO and the European Union have set MRL for spiromesifen and spirodiclofen in tomato fruit to protect consumer health. To assist the Egyptian government in developing regulations for the tested pesticides, the chronic risk quotient (RQ_c_) was determined for adults. Field data were used to assess dietary risk by comparing the national estimated daily intake (NEDI) with the appropriate acceptable daily intake (ADI) [39,40]. The median residue levels (STMR) for spiromesifen were 0.412 mg/kg and 0.635 mg/kg, using authorized and double doses, respectively. The calculated NEDI values for spiromesifen were 1.47E-03-2.26E-03 mg/kg.bw/day, while the RQ_c_ values were 4.89–7.54%, well below 100. The STMR of spirodiclofen was 0.398 mg/kg and 0.471 mg/kg, respectively. The NEDI values for spirodiclofen were determined to be 1.42E-03-1.68E-03 mg/kg.bw/day, and RQ_c_ values ranged from 9.46 to 11.19 %, which were well below 100 (Table 6), indicating that spiromesifen and spirodiclofen at the authorized and double authorized doses for tomato fruit do not pose a health risk.

## 3. Materials and Methods

### 3.1. Chemicals and Solutions

Reference standards of spirodiclofen and spiromesifen with purities of 98.9% and 99.5%, respectively, were purchased from Dr. Ehrenstorfer (Augsburg, Germany). HPLC-grade acetonitrile and methanol and LC-MS-grade formic acid were provided by Fisher Scientific (Loughborough, UK). Primary secondary amine (PSA) was supplied by Agilent Technologies (Santa Clara, CA, USA). Anhydrous magnesium sulfate (purity >98%) and sodium chloride were purchased from Chem-Lab NV (Zedelgem, Belgium). An Ultra Clear™ system from Evoqua Water Technologies LLC (Günzburg, Germany) produced ultrapure water. Commercial formulations of spiromesifen (24%, suspended concentration, SC) and spirodiclofen (24%, suspended concentration, SC) (Bayer Crop Science AG, Monheim am Rhein, Germany) were purchased from the local market.

### 3.2. Standard Solutions Preparation

Stock solutions of spiromesifen and spirodiclofen (1000 mg/L) were prepared individually by dissolving the appropriate amount in acetonitrile. A mixture of intermediate and working standard solutions of spiromesifen and spirodiclofen of 100 and 10 mg/L was prepared by further dilution. The matrix-matched calibration curves were prepared in a tomato blank previously prepared according to the proposed analytical approach. The standard solutions were stored at −20 °C.

### 3.3. Field Trial

Field experiments were carried out under Egyptian field conditions in El-Salheya El Gedida, Sharqia Governorate. Commercial formulations of spiromesifen (24%, SC) and spirodiclofen (24%, SC) were sprayed at the manufacturer’s authorized dose of 84 and 120 g a.i/ha and at a double dose of 168 and 240 g a.i/ha, respectively. The spraying was conducted using a knapsack sprayer, and 1000 L ha^−1^ of tap water was used for dilution. To avoid cross-contamination, the experimental field was divided into fifteen 50 m^2^ plots; three were designated as blank plots (no treatments), and the others were divided into three plots for each treatment. The temperature during the experiment ranged from 27 to 34 °C during the day and from 20 to 23 °C at night; the humidity ranged from 40 to 52%, and the average sunshine duration was about 11 h. Representative samples of tomatoes (about 2 kg) were randomly collected at different harvests within 14 days after spraying, then immediately transported to the laboratory, cut into pieces, homogenized in HOBART food processor (Troy, OH, USA), and stored at −20 °C.

### 3.4. Terminal Residues

For determination of terminal residues of spiromesifen and spirodiclofen, both insecticides were applied individually 2 or 3 times in 7-day intervals using the authorized dose of 84 and 120 g a.i/ha, or at a double dose of 168 and 240 g a.i/ha, respectively. Samples were randomly taken from the sprayed plots on the 3rd and 7th day after the second and/or third treatment.

### 3.5. Sample Extraction

First, 10 ± 0.1 g of the homogenized frozen sample was weighed into a 50 mL centrifuge tube. Then, 10 mL of acetonitrile was added, followed by a ceramic homogenizer. The tube was shaken for 1 min. For salting out, 5 g of a salt mixture consisting of 4 g magnesium sulfate and 1 g sodium chloride was added, and the tube was again shaken for 30 s before centrifugation at 5000 rpm for 3 min. A portion of the top layer was filtered through a 0.22-micron syringe filter and then diluted 8-fold with acetonitrile for LC-MS/MS analysis.

### 3.6. LC-MS/MS

For chromatographic analysis, a Dionex Ultimate 3000 RS UHPLC system separation module was combined with a TSQ Altis triple quadrupole mass spectrometer (MS/MS) (Thermo Fisher Scientific, Austin, TX, USA). An Accucore RP-MS C18 column (100 × 2.1 mm, 2.6-micrometer particle size) was used to separate analytes at 40 °C. The mobile phase consisted of 100% ultrapure water (A) and 100% methanol (B). The mobile phase gradient program started at 40% B for 1 min, was increased to 95% B over 3 min (1–4 min), then held for 5 min (4–9 min), and returned to the initial 40% B over 0.1 min (9–9.1 min), which was then held for 6 min, giving a total run time of 16 min. The injection volume was 5 µL, and the elution flow rate was 0.3 mL/min. The MS detection was performed in multiple reaction monitoring (MRM) mode. The ion spray voltage was set at 3800 V, the ion transfer tube temperature was set at 325 °C, and the vaporizer temperature was set at 350 °C. The auxiliary and sheath gasses pressures were set to 10 and 40 arb, respectively. Control of the system and data acquisition were performed using the Trace Finder program (version 4.1).

### 3.7. Method Validation

The criteria of linearity range, limits of quantification (LOQ) and determination (LOD), recovery percent, accuracy, and matrix effects (ME) were assessed in accordance with the requirements of the SANTE/12682/2019 guideline [25]. A blank sample extract was used to assess the absence of interference with the corresponding analytes’ times of retention (selectivity). The squared correlation coefficient (R^2^) and the relative residuals were used to evaluate the linearity range for the calibration curves constructed in solvent or blank matrix, and the obtained slopes were compared to determine matrix effects. The LOD was determined based on the ratios of signal to noise (S/N = 3)

The lower concentration at which the method showed satisfactory recovery of 70–120% and precision of ≤20% is known as the limit of quantitation (LOQ). The recoveries were assessed at four spiking levels of 0.01, 0.1, 1, and 5 mg/kg. Method accuracy was evaluated at the level of concentration of 0.01 mg/kg by assessing the precision and trueness (% recovery); precision was reported as relative standard deviation (RSD) of the recovered sample analytes spiked at 0.01 mg/kg within-day (RSD_r_, n = 6, in one day) and between-days (RSD_R_, n = 18, on three different days) repeatability.

### 3.8. Statistic Calculations

The Microsoft Excel 2021 program was used for the calculations of field results and for the study of significance between groups by performing the one-way ANOVA test and Fisher’s least significant difference (LSD), where a probability value of *p* < 0.05 was considered significant.

The first-order kinetic model was used to describe the dissipation rate of spiromesifen and spirodiclofen residues in tomato field samples using the exponential formula of C_t_ = C_o_ e^-kt^, where C_t_ represents the concentration in mg/kg at time t (days), C_o_ represents the concentration at 0 days, and k represents the degradation rate constant (days^−1^). The half-life was determined using Hoskins formula: t*_1/2_* = In2/k [39]; the pre-harvest interval (PHI) was determined by the formula PHI = Ln (MRL/C_0_)/k [40,41].

### 3.9. Dietary Risk Assessment

The terminal residue data from the field experiment were used to assess the long-term risk (chronic risk) upon exposure to spiromesifen and spirodiclofen by comparing the national estimated daily intake (NEDI) to the acceptable daily intake (ADI) (mg/kg.bw) (RQc = NEDI/ADIx100) [42,43]. The national estimated daily intake was calculated as NEDI = Fi × STMRi/bw, where Fi represents the dietary intake (0.214 kg/day) according to the WHO Cluster Diet G06 [44], STMRi is supervised trials median residue (mg/kg), and bw represents the adult body weight (60 kg) [45]. The acceptable daily intake (ADI) for spiromesifen and spirodiclofen is 0.03 and 0.5 mg/kg.bw [46,47]. It is generally accepted that RQ > 100 is considered an unacceptable risk to humans, while RQ < 100 is considered a minimal risk [48,49].

## 4. Conclusions

In this study, a rapid, simple, and accurate procedure for determining spiromesifen and spirodiclofen residues in tomato fruit was optimized and validated. The method showed excellent linearity, recoveries, and precision. The levels of spiromesifen and spirodiclofen in tomato fruit samples from field trials were analyzed. The results show that spiromesifen and spirodiclofen readily decline in tomato fruit with a half-life of 1.49–1.83 and 1.9–2.38 days, respectively. Final residue levels of spiromesifen and spirodiclofen were lower than the MRL of 1 and 0.5 mg/kg set by the European Union regulations, respectively, 7 days after application of the authorized dose and double the authorized dose two or three times. The RQc values were below 100, and neither poses a risk to human health. These research results will assist the Egyptian government in formulating guidelines for the safe and proper use of spiromesifen and spirodiclofen on tomato fruits grown under open field conditions.

## Figures and Tables

**Table 1:** MS/MS parameters for determination of spiromesifen and spirodiclofen.

Analyte	Precursor Ion [M + H]^+^	Product Ions (*m/z*)	Collision Energy (V)	RF Lens (V)	Dwell Time (ms)	Rt (min)
Spiromesifen	371.2	255.2	31	76	148	8.09
	371.2	***273.2***	11	76	148	
Spirodiclofen	411.2	71.07	16	60	148	8.24
	411.2	***313.2***	10	60	148	

The bold italic ions were used as quantifier ions.

**Table 2:** Calibration parameters, LOD, LOQ, ME (%), and accuracy of spiromesifen and spirodiclofen in tomato fruits.

Analyte	Linear Range (µg/kg)	R^2^	Residual (%)	LOD (µg/kg)	LOQ (µg/kg)	ME (%)	Accuracy			
							Intra-Day (n = 6)		Inter-Days (n = 18)	
							R (%)	RSD_r_ (%)	R (%)	RSD_R_ (%)
Spiromesifen	1–100	0.9993	16.4	0.26	5	−8.9	94.22	8.35	96.42	15.85
Spirodiclofen	0.5–100	0.9991	13.2	0.08	5	−7.2	91.34	6.68	92.64	11.64

**Table 3:** Recovery and RSD of spiromesifen and spirodiclofen in tomato fruits.

Analyte	Recovery ± RSD (%), (n = 6)			
	0.01 mg/kg	0.10 mg/kg	1 mg/kg	5 mg/kg
Spiromesifen	93.52 ± 6.22	90.75 ± 4.31	95.64 ± 3.14	97.22 ± 8.78
Spirodiclofen	89.23 ± 5.87	94.13 ± 9.11	92.55 ± 12.88	96.71 ± 7.33

**Table 4:** Dissipation kinetics of spiromesifen and spirodiclofen in tomato fruits.

	Spiromesifen		Spirodiclofen	
	Dosage (g a.i/ha)		Dosage (g a.i/ha)	
	120	240	84	168
Slope (C*_0_*) (mg/kg)	3.159	4.308	2.126	3.365
Intercept (k)	0.463	0.379	0.362	0.291
t*_1/2_* (days)	1.49	1.83	1.91	2.38
r^2^	0.9904	0.972	0.9915	0.996
PHI (days)	2.48	3.85	3.99	6.55

**Table 5:** Terminal residue of spiromesifen and spirodiclofen in tomato fruits.

	Dosage (g a.i/ha)	Spray Times	Interval (Days)	Residue * (mg/kg) ± SD
Spiromesifen	120	2	3	0.798 ± 0.211
			7	0.352 ± 0.107
		3	3	0.471 ± 0.166
			7	0.168 ± 0.084
	240	2	3	1.652 ± 0.327
			7	0.757 ± 0.143
		3	3	1.412 ± 0.225
			7	0.307 ± 0.119
Spirodiclofen	84	2	3	0.585 ± 0.314
			7	0.101 ± 0.112
		3	3	0.772 ± 0.221
			7	0.281 ± 0.068
	168	2	3	1.414 ± 0.345
			7	0.398 ± 0.117
		3	3	0.962 ± 0.253
			7	0.291 ± 0.138

* Residue is the mean of three replicates collected from three different plots.

**Table 6:** Supervised trials median residue (STMR), national estimated daily intakes (NEDI), and chronic risk quotient (RQ_c_) of spiromesifen and spirodiclofen in tomato fruits.

	Dosage (g a.i/ha)	STMR (mg/kg)	NEDI (mg/kg bw/d)	RQ_c_ (%)
Spiromesifen	84	0.4115	1.47E-03	4.89
	168	1.0845	3.87E-03	12.89
Spirodiclofen	120	0.433	1.54E-03	10.30
	240	0.68	2.43E-03	16.17

## Data Availability

All data will be available upon reasonable request from the corresponding author.

## References

[1] (2019). FAOSTAT; Food and Agriculture Organization of the United Nations. http://www.fao.org/faostat/en/#data/QC.

[2] Beecher G.R. (1998). Nutrient content of tomatoes and tomato products. Proc. Soc. Exp. Biol. Med..

[3] Shi J., Maguer M.L. (2000). Lycopene in tomatoes: Chemical and physical properties affected by food processing. Crit. Rev. Food Sci. Nutr..

[4] Sies H., Stahl W., Sundquist A.R. (1992). Antioxidant Functions of Vitamins: Vitamins E and C, Beta-Carotene, and Other Carotenoids a. Ann. N. Y. Acad. Sci..

[5] Friedman M. (2013). Anticarcinogenic, cardioprotective, and other health benefits of tomato compounds lycopene, α-tomatine, and tomatidine in pure form and in fresh and processed tomatoes. J. Agric. Food Chem..

[6] Olaniyi J., Akanbi W., Adejumo T., Ak O. (2010). Growth, fruit yield and nutritional quality of tomato varieties. Afr. J. Food Sci..

[7] Schmidt-Jeffris R., Snipes Z., Bergeron P. (2021). Acaricide efficacy and resistance in South Carolina tomato populations of twospotted spider mite. Fla. Entomol..

[8] Khan N., Yaqub G., Hafeez T., Tariq M. (2020). Assessment of Health Risk due to Pesticide Residues in Fruits, Vegetables, Soil, and Water. J. Chem..

[9] Dekeyser M.A. (2005). Acaricide mode of action. Pest Manag. Sci..

[10] Varghese T.S., Mathew T.B., George T., Beevi S.N., Xavier G. (2011). Dissipation of propargite and spiromesifen in/on chilli fruits. Pestic. Res. J..

[11] Raj M., Solanki P., Singh S., Vaghela K., Shah P., Patel A., Diwan K. (2012). Dissipation of spiromesifen in/on okra under middle Gujarat conditions. Pestic. Res. J..

[12] Siddamallaiah L., Mohapatra S. (2016). Residue level and dissipation pattern of spiromesifen in cabbage and soil from 2-year field study. Environ. Monit. Assess..

[13] Sharma K., Dubey J., Mukherjee I., Parihar N., Battu R., Singh B., Sharma I. (2006). Residual behavior and risk assessment of Spiromesifen (Oberon 240 SC) on eggplant (Solanum melonongena L) in India: A multilocational study. Bull. Environ. Contam. Toxicol..

[14] Siddamallaiah L., Mohapatra S., Buddidathi R., Hebbar S.S. (2017). Dissipation of spiromesifen and spiromesifen-enol on tomato fruit, tomato leaf, and soil under field and controlled environmental conditions. Environ. Sci. Pollut Res..

[15] Sun H., Liu C., Wang S., Liu Y., Liu M. (2013). Dissipation, residues, and risk assessment of spirodiclofen in citrus. Environ. Monit. Assess..

[16] Sun D., Zhu Y., Pang J., Zhou Z., Jiao B. (2016). Residue level, persistence and safety of spirodiclofen–pyridaben mixture in citrus fruits. Food Chem..

[17] Fernández-Alba A.R., Tejedor A., Agüera A., Contreras M., Garrido J. (2000). Determination of imidacloprid and benzimidazole residues in fruits and vegetables by liquid chromatography–mass spectrometry after ethyl acetate multiresidue extraction. J. AOAC Int..

[18] Luke M.A., Froberg J.E., Doose G.M., Masumoto H.T. (1981). Improved multiresidue gas chromatographic determination of organophosphorus, organonitrogen, and organohalogen pesticides in produce, using flame photometric and electrolytic conductivity detectors. J. Assoc. Off. Anal. Chem..

[19] Andersson A., Palsheden H. (1981). Comparison of the efficiency of different GLC multi-residue methods on crops containing pesticide residues. Fresenius J. Anal. Chem..

[20] Wilkowska A., Biziuk M. (2011). Determination of pesticide residues in food matrices using the QuEChERS methodology. Food Chem..

[21] Hou X., Han M., Dai X., Yang X., Yi S. (2013). A multi-residue method for the determination of 124 pesticides in rice by modified QuEChERS extraction and gas chromatography—Tandem mass spectrometry. Food Chem..

[22] Anastassiades M., Lehotay S.J., Štajnbaher D., Schenck F.J. (2003). Fast and easy multiresidue method employing acetonitrile extraction/partitioning and “dispersive solid-phase extraction” for the determination of pesticide residues in produce. J. AOAC Int..

[23] Song N.E., Yoo M., Nam T.G. (2019). Multi-residue analysis of 203 pesticides in strawberries by liquid chromatography tandem mass spectrometry in combination with the QuEChERS method. CyTA J. Food.

[24] Lehotay S. (2007). pesticide residues in foods by acetonitrile extraction and partitioning with Magnesium Sulfate. J. AOAC Int..

[25] SANTE/12682/2019. Guidance Document on Analytical Quality Control and Method Validation Procedures for Pesticides Residues Analysis in Food and Feed. https://ec.europa.eu/food/sites/food/files/plant/docs/pesticides_mrl_guidelines_wrkdoc_2019-12682.pdf.

[26] Abdallah O.I., Ahmed N.S. (2019). Development of a vortex-assisted dispersive liquid-liquid microextraction (VA-DLLME) and LC-MS/MS procedure for simultaneous determination of fipronil and its metabolite fipronil sulfone in tomato fruits. Food Anal. Methods.

[27] Ferrer C., Lozano A., Agüera A., Girón A.J., Fernández-Alba A. (2011). Overcoming matrix effects using the dilution approach in multiresidue methods for fruits and vegetables. J. Chromatogr. A.

[28] Kaczyński P. (2017). Clean-up and matrix effect in LC-MS/MS analysis of food of plant origin for high polar herbicides. Food Chem..

[29] Li Y., Lu P., Hu D., Bhadury P.S., Zhang Y., Zhang K. (2015). Determination of dufulin residue in vegetables, rice, and tobacco using liquid chromatography with tandem mass spectrometry. J. AOAC Int..

[30] Dubey J.K., Katna S., Shandil D., Devi N., Singh G., Singh G., Sharma A. (2021). Dissipation kinetics and dietary risk assessment of spiromesifen on major summer vegetables using good agricultural practices. Biomed. Chromatogr..

[31] Sharma K., Mukherjee I., Sing B., Mandal K., Sahoo S.K., Banerjee H., Patel H.K. (2014). Persistence and risk assessment of spiromesifen on tomato in India: A multilocational study. Environ. Monit. Assess..

[32] Vinothkumar B., Shibani P., Kaviya R., Kowshika J. (2018). Dissipation pattern of spiromesifen in/on brinjal fruits. Int. J. Chem. Stud..

[33] Sharma K., Dubey J., Kumar A., Gupta P., Singh B., Sharma I., Nath A. (2005). Persistence and Safety Evaluation of Spiromesifen on Apple (Maius domestica L) in India: A Multilocation Study. Pestic. Res. J..

[34] Sharma K., Dubey J., Deka S., Chandrasekaran S., Gupta P., Kumar A., Kennedy J. (2007). Dissipation kinetics of spiromesifen on tea (Camellia sinensis) under tropical conditions. Chemosphere.

[35] Sharma K., Rao C.S., Dubey J., Patyal S., Parihar N., Battu R., Jaya M. (2007). Persistence and dissipation kinetics of spiromesifen in chili and cotton. Environ. Monit. Assess..

[36] Wang Y., Deng Y., Li Q., Wu J., Yang X., Wu F., Qin Y. (2020). Degradation of Spirodiclofen and Spirotetramat Residue in Kumquat with Film Mulching. Chin. J. Trop. Crops.

[37] Amany R.M., Dalia E.E. (2017). Residues assessment of captan, spirodiclofen and thiophanate-methyl in apple fruits under the field conditions. Middle East J. Agric. Res..

[38] Fantke P., Juraske R. (2013). Variability of pesticide dissipation half-lives in plants. Environ. Sci. Technol..

[39] Hoskins W. (1961). Mathematical treatment of the rate of loss of pesticide residues. FAO Plant Prot. Bull..

[40] Hingmire S., Oulkar D.P., Utture S.C., Shabeer T.A., Banerjee K. (2015). Residue analysis of fipronil and difenoconazole in okra by liquid chromatography tandem mass spectrometry and their food safety evaluation. Food Chem..

[41] Abdallah O.I., Soliman H., El-Hefny D., Abd El-Hamid R., Malhat F. (2021). Dissipation profile of sulfoxaflor on squash under Egyptian field conditions: A prelude to risk assessment. Int. J. Environ. Anal. Chem..

[42] Brancato A., Brocca D., Ferreira L., Greco L., Jarrah S., Leuschner R., Nougadere A. (2018). Use of EFSA pesticide residue intake model (EFSA PRIMo revision 3). EFSA J..

[43] Malhat F., Abdallah O.I. (2019). Residue distribution and risk assessment of two macrocyclic lactone insecticides in green onion using micro-liquid-liquid extraction (MLLE) technique coupled with liquid chromatography tandem mass spectrometry. Environ. Monit. Assess..

[44] (2013). WHO/GEMS/FOODS. https://www.who.int/data/gho/samples/food-cluster-diets.

[45] FAO (2009). Manual on the Submission and Evaluation of Pesticide Residues Data.

[46] EFSA (2012). Conclusion on the peer review of the pesticide risk assessment of the active substance spiromesifen. EFSA J..

[47] Bellisai G., Bernasconi G., Brancato A., Cabrera L.C., Ferreira L., Kazocina A. (2021). Review of the existing maximum residue levels for spirodiclofen according to Article 12 of Regulation (EC) No 396/2005. EFSA J..

[48] Malhat F., Boulangé J., Abdelraheem E., Abdallah O.I., Abd El-Hamid R., Abd El-Salam S. (2017). Validation of QuEChERS based method for determination of fenitrothion residues in tomatoes by gas chromatography–flame photometric detector: Decline pattern and risk assessment. Food Chem..

[49] Li R., Liu T., Cui S., Zhang S., Yu J., Song G. (2017). Residue behaviors and dietary risk assessment of dinotefuran and its metabolites in Oryza sativa by a new HPLC–MS/MS method. Food Chem..

